# Mutational analysis of *AGXT* in two Chinese families with primary hyperoxaluria type 1

**DOI:** 10.1186/1471-2369-15-92

**Published:** 2014-06-17

**Authors:** Guo-min Li, Hong Xu, Qian Shen, Yi-nv Gong, Xiao-yan Fang, Li Sun, Hai-mei Liu, Yu An

**Affiliations:** 1 Children's Hospital of Fudan University, 399 Wanyuan Road, Minhang District, Shanghai 201102, China; 2 Institutes of Biomedical Sciences of Fudan University, 120 Dongan Road, Xuhui District, Shanghai 200023, China

**Keywords:** *AGXT* gene, Chinese children, Mutational analysis, Novel mutation, Primary hyperoxaluria type 1

## Abstract

**Background:**

Primary hyperoxaluria type 1 is a rare autosomal recessive disease of glyoxylate metabolism caused by a defect in the liver-specific peroxisomal enzyme alanine:glyoxylate aminotransferase (AGT) that leads to hyperoxaluria, recurrent urolithiasis, and nephrocalcinosis.

**Methods:**

Two unrelated patients with recurrent urolithiasis, along with members of their families, exhibited mutations in the *AGXT* gene by PCR direct sequencing.

**Results:**

Two heterozygous mutations that predict truncated proteins, p.S81X and p.S275delinsRAfs, were identified in one patient. The p.S81X mutation is novel. Two heterozygous missense mutations, p.M1T and p.I202N, were detected in another patient but were not identified in her sibling. These four mutations were confirmed to be of paternal and maternal origin.

**Conclusions:**

These are the first cases of primary hyperoxaluria type 1 to be diagnosed by clinical manifestations and *AGXT* gene mutations in mainland China. The novel p.S81X and p.I202N mutations detected in our study extend the spectrum of known *AGXT* gene mutations.

## Background

Primary hyperoxaluria type 1 (PH1; MIM# 259900) is an autosomal recessive disorder of glyoxylate metabolism, leading to the overproduction of endogenous oxalate; patients present with urolithiasis and/or nephrocalcinosis [1,2]. The disease is caused by mutations in the *AGXT* gene (MIM#604285), which encodes the hepatic peroxisomal enzyme alanine:glyoxylate aminotransferase (AGT; EC 2.6.1.44), a pyridoxal 5′-phosphate (PLP)-dependent enzyme that catalyses the transamination of glyoxylate to glycine [3,4]. Approximately 50% of patients who present with PH1 in childhood will have end-stage renal failure by the age of 15 years. PH1 is a condition that leads to systemic oxalosis with oxalate precipitation in the eye, heart, and bones and that results in significant morbidity and mortality [5,6]. The incidence of PH1 is estimated at 1 in 120,000 live births with prevalence ranging from 1.05/10^6^ to 2.9/10^6^ in France, Switzerland, and the Netherlands [5,7-9]. Excessive oxalate excretion is an indicator of this disease; however, the test is not specific for PH1 and may have misleading results because oxalate excretion may be reduced during renal failure [2,10,11]. Thus, more sophisticated tests, including genetic analysis and/or enzymology, are required for diagnosis [12-14]. Although a few sporadic cases of PH1 have been reported in mainland China, mutational analysis of *AGXT* was not performed in these cases. We identified 2 unrelated cases of PH1 in mainland China. We analysed the clinical features, detected *AGXT* mutations in their families, and compared our cases with other ethnic or regional patients previously reported by other authors. We hope that the presentation of rare cases will contribute to understanding the spectrum of the disease by aiding its clinical identification and pathogenetic understanding.

## Methods

### Patients

**Figure 1:**
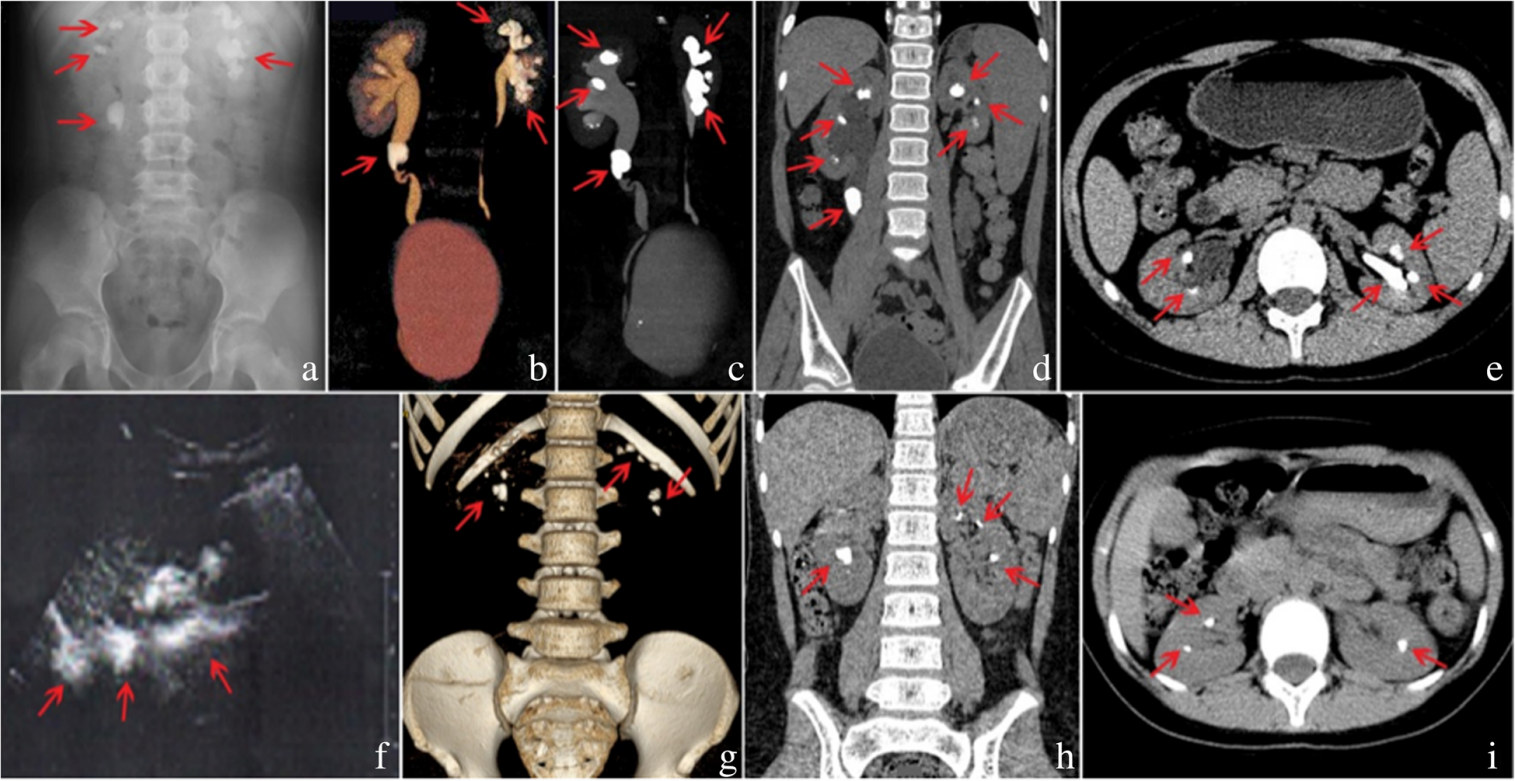
**Radiographic findings in patient 1 (a-e) and patient 2 (f-i).** Arrows indicate stones. **a**: abdominal X-ray, **b**: CT (coronal image), **c**: VR (volume rendering), **d**: MIP (maximum intensity projection), **e**: CT (axial image), **f**: ultrasound, **g**: VR (volume rendering), **h**: CT (coronal image), **i**: CT (axial image).

PH1 was diagnosed in two patients from two unrelated Chinese families. Patient 1, a 10-year-old girl, presented with gross haematuria at the age of 3. She went to her local hospital, and a renal ultrasound showed several small stones in the kidneys and no hydronephrosis. Urologists at the local hospital administered conservative treatment (i.e., high fluid intake), and her haematuria gradually disappeared. The patient presented with gross haematuria again at the age of 5, this time with abdominal pain. A renal ultrasound displayed several stones in the kidneys, a stone (6 mm × 9 mm) in the right ureter, and right hydronephrosis. An abdominal plain film X-ray also revealed multiple urolithiasis (Figure 1a). Urologists at the local hospital administered extracorporeal shock wave lithotripsy (ESWL). After ESWL, the patient excreted several stones in her urine, and her gross haematuria and back pain resolved. The patient developed right back pain again at age 10. A renal ultrasound indicated multiple urolithiasis, a stone (5 mm × 9 mm) in the right ureter, and right hydronephrosis. Renal function tests suggested elevated levels of both plasma creatinine (185 μmol/L) and blood urea nitrogen (BUN, 11.10 mmol/L). Urologists at the local hospital again administered ESWL. After ESWL, her back pain was remissive, but her renal function remained abnormal. Therefore, the patient was admitted to our centre (the Centre of Nephrology and Urology, Children’s Hospital of Fudan University) for evaluation. Her parents had no consanguinity, and there was no family history of renal disease.

On admission, a physical examination showed normal parameters. Her height was between the 25th and 50th percentile, and her body weight was in the 50th percentile. Laboratory tests indicated normal haemoglobin (11.9 g/100 ml) and renal insufficiency (filtration rate < 45 ml/min/1.73 m^2^, creatinine level 195 μmol/L, and BUN 13.20 mmol/L). Blood gases revealed mild metabolic acidosis (pH 7.28, BE 3.5 mmol/L), and constituent analysis suggested that the stones were composed of calcium oxalate monohydrate (>95%). An abdominal computed tomography (CT) scan, CT urography, and three-dimensional image of CT urography all showed multiple urolithiasis (Figure 1b,c,d, and e). A CT urography and three-dimensional image of CT urography also revealed right hydronephrosis (Figure 1c and d). Based on the clinical data, PH1 and chronic kidney disease stage 3 (CKD 3) were diagnosed. The patient received conservative treatment, including a high fluid intake, oral potassium citrate, and vitamin B6 (pyridoxine). The patient progressed to CKD 5 within 7 months, after which she received haemodialysis three times a week. The adequacy of haemodialysis was good (Kt/v 1.49, urea clearance 70%). The levels of calcium and phosphorus in the blood were normal, and parathyroid hormone levels were between 280 to 320 pg/L.

The parents had been clinically evaluated as normal at the affiliated Obstetrics and Gynaecology Hospital of Fudan University. The parents were experiencing their second pregnancy (20 weeks gestation) and were referred to our centre for prenatal genetic diagnosis.Patient 2, a 7-year-old girl, presented with gross haematuria and abdominal pain at the age of 5. She went to her local hospital, and a renal ultrasound revealed urolithiasis. Urologists at the local hospital administered conservative treatment, including a high fluid intake and herbal medicine. After treatment, the patient excreted several stones in the urine, and her symptoms disappeared. The patient developed gross haematuria and abdominal pain again at the age of 6, and a renal ultrasound showed urolithiasis and right hydronephrosis (Figure 1f). Urologists at the local hospital continued to administer conservative treatment because the stones in the kidneys were small and stones were periodically excreted in her urine. At 7 years of age, the patient was referred to our centre for evaluation. Her parents had no consanguinity, and there was no family history of renal disease.

On admission, a physical examination indicated normal parameters. Her height was between the 75th and 90th percentile, and her body weight was in the 75th percentile. Laboratory tests revealed haematuria (8 RBCs/HPF), normal haemoglobin (12.1 g/100 ml), and normal renal function (filtration rate <90 ml/min/1.73 m^2^, creatinine level 49.0 mmol/L, and BUN 3.7 mmol/L). The blood gases were normal (pH 7.35, BE 1 mmol/L), and the parathyroid hormone level was normal. Constituent analysis also suggested that the stones were made of calcium oxalate monohydrate (>95%). An abdominal CT scan indicated multiple urolithiasis (Figure 1g,h, and i). Based on the clinical data, the patient was diagnosed with PH1. The patient received conservative treatment, such as a high fluid intake, oral potassium citrate, and vitamin B6 (pyridoxine). The patient's condition is currently being followed in our centre, and her renal function is normal.

The medical histories of the two families were also investigated. Informed consent was obtained from the parents. Ethical committee approval was obtained from the Ethical Committee of Children’s Hospital of Fudan University.

### Mutation analysis of the *AGXT* gene

Genomic DNA was extracted from the members of both families and purified from peripheral leukocytes in whole-blood samples and cast-off cells in amniotic fluid using a DNA isolation kit. All *AGXT* exons were amplified by polymerase chain reaction (PCR). The *AGXT* primers were designed on the basis of previously published information regarding intron-exon boundaries [15,16]. The PCR products were purified with a QIAquick PCR Purification Kit (Qiagen, Hilden, Germany). The purified products were cycle-sequenced with Big Dye terminators (Applied Biosystems, Foster City, CA, USA), and the cycle sequence products were analysed with an automated sequencer (ABI Prism 310 Genetic Analyser). Novel *AGXT* mutations were investigated in 100 healthy controls by direct sequencing.

### *In silico* prediction of amino-acid substitution

Nonsynonymous sequence variants of genes were analysed with the PolyPhen-2 software [17] and SIFT algorithm [18] to predict the impact of the amino-acid substitution. PolyPhen-2 calculates a Naive Bayes posterior probability that any mutation is damaging by the representation of a score ranging from 0 to 1 and predicts qualitative damage based on the model’s false positive rate (benign, possibly damaging, or probably damaging). The SIFT prediction is based on the degree of conservation of amino-acid residues in sequence alignments derived from closely related sequences, which also provides a prediction of qualitative damage (tolerated or damaging).

### Web resources

The following GenBank sequences ( http://www.ncbi.nlm.nih.gov/Genbank/) served as reference files: NT_005416 for *AGXT* genomic nucleotide position and NM_000030.2 for *AGXT* cDNA position. Uni-ProtKB ( http://www.uniprot.org/uniprot/P21549, ID: P21549) and Ensembl Genome Browser ( http://www.ensembl.org, ID: ENST00000307503) were used to determine the AGXT amino-acid position, and Clustal W ( http://www.ebi.ac.uk/clustalw/) was used for multiple sequence alignment.

## Results

### Clinical features

Two girls presented with recurrent and multiple urolithiasis and were diagnosed with PH1. Patient 1 progressed to end-stage renal disease at the age of 10.6 years. The renal function of patient 2 is currently normal. Renal ultrasounds and urinary analyses were both normal in other members of the families. The clinical features are summarised in Table 1.

**Table 1:** **Clinical features and *AGXT *** **mutations in two patients with PH1**

**Case**	**Gender**	**Age at onset (Y)**	**Age at progression to ESRD (Y)**	**Main symptoms**	**Family history**	**Radiological findings**	**Mutation**	**Mutation origin**	**Type of mutation**
1	Female	3	10.6	Recurrent urolithiasis	No	Multiple urolithiasis	p.S81X	Mother	Het
							p.S275delinsRAfs	Father	Het
2	Female	5	-	Recurrent urolithiasis	No	Multiple urolithiasis	p.M1T	Father	Het
							p.I202N	Mother	Het

-:no progression to ESRD.

### Mutational analysis of the *AGXT* gene

#### Family 1

**Figure 2:**
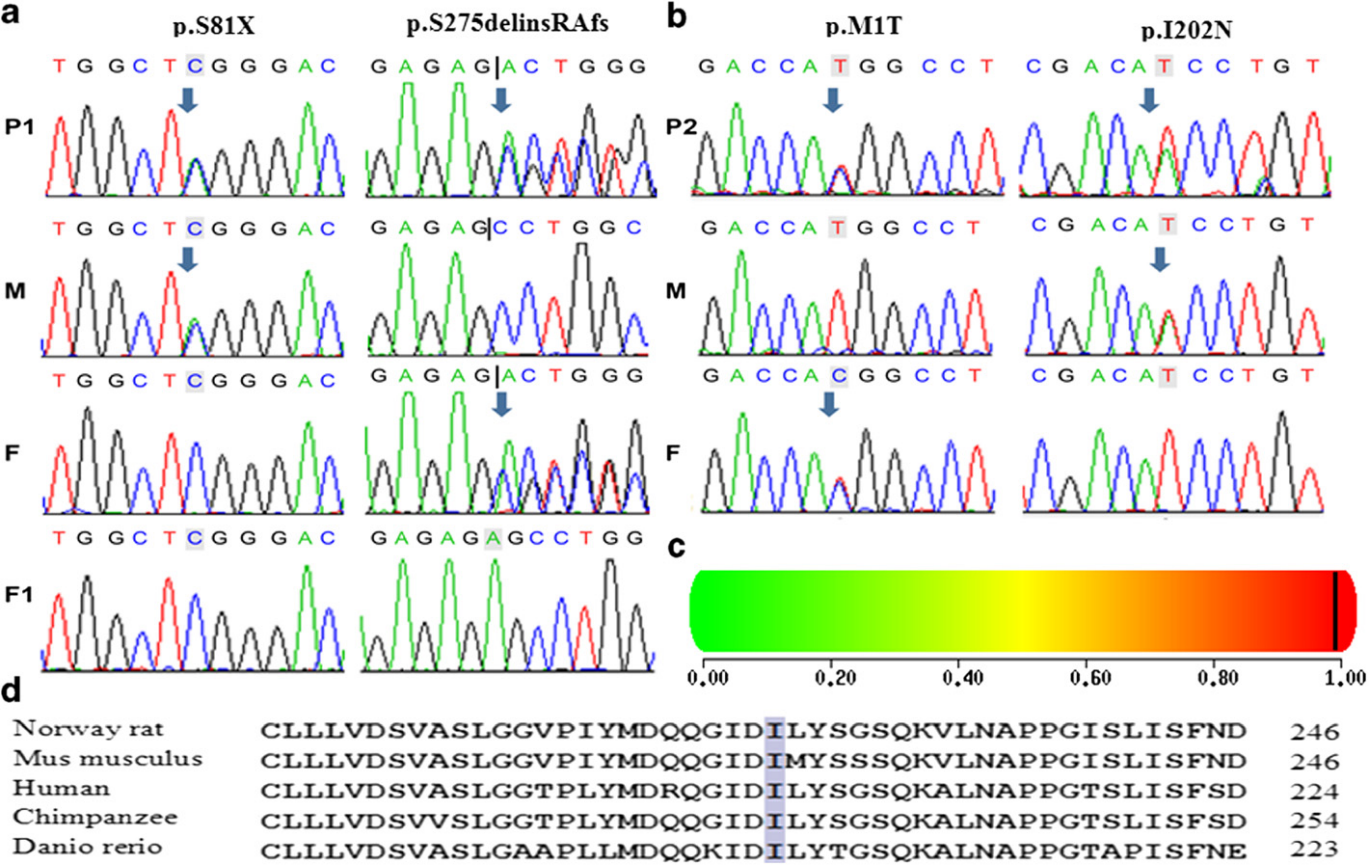
***AGXT *gene mutational analysis.** Two mutations, p.S81X and p.Ser275delinsRAfs, in patient 1 **(a)**. Two mutations, p.M1T and p.I202N, in patient 2 **(b)**. PolyPhen-2 predicted the p.I202N mutation to be a “damaging mutation” with a score of 0.968 (sensitivity: 0.77; specificity: 0.95) **(c)**. Alignment of the mutated p.I202N AGT protein with different species shows the complete conservation of the amino acid in grey **(d)**. Arrows indicate the mutation sites. P1: patient 1, P2: patient 2, F: father, M: mother, F1: foetus.

Two heterozygous mutations, p.S81X (c.242C > A) in exon 2 and p.S275delinsRAfs (c.823_824dupAG) in exon 7, were identified in patient 1, and these mutations were inherited from each parent. The p.S275delinsRAfs mutation has been previously reported as a disease-causing mutation. An additional nonsense mutation of p.Ser81X that creates a truncated AGT is novel and was not identified in 100 healthy controls; this mutation is hypothesised to be pathogenic. A recessive genetic model of the compound heterozygous mutations of *AGXT* in patient 1 indicated the cause of PH1. After the mutations were confirmed in family 1, *AGXT* mutational analysis was then performed for the foetus using an amniotic fluid sample for prenatal genetic diagnosis. This analysis revealed no mutations in the exons, indicating that the nonsense p.S81X mutation and the frameshift p.S275delinsRAfs mutation (Figure 2a) were not inherited.

#### Family 2

Two heterozygous missense mutations, p.M1T (c.2 T > C) in exon 1 and p.I202N (c.605 T > A ) in exon 6, were detected in patient 2 (Figure 2b), and these mutations were inherited from each parent. The p.M1T mutation has been previously reported, whereas the p.I202N mutation is novel and was not identified in 100 healthy controls. PolyPhen-2 predicts the pathogenicity of p.I202N to be “Probably Damaging” with a score of 0.968 (sensitivity: 0.77; specificity: 0.95) (Figure 2c); SIFT software predicted this mutation to be harmful, with a score of 0.03. In addition, multiple sequence alignments revealed that the p.I202N mutation occurs in a residue that is highly evolutionarily conserved among humans, Norway rats, *Mus musculus*, *Danio rerio* and chimpanzees (Figure 2d).

## Discussion

PH1 is a severe autosomal recessive inherited disorder of glyoxylate metabolism caused by mutations in the *AGXT* gene on chromosome 2q37.3 that encodes the liver-specific PLP-dependent enzyme AGT [4]. More than 150 different pathogenic mutations, including nonsense, frameshift, and missense mutations, in the *AGXT* gene that cause PH1 have been identified to date ( http://www.hgmd.cf.ac.uk/ac/gene.php?gene=AGXT). While nonsense and frameshifts are null mutations that lead to the complete loss of the gene product, the most common type of *AGXT* mutations are single amino-acid substitutions that lead to the synthesis of an aberrant gene product [6]. These mutations are found throughout the entire gene and cause a wide spectrum of clinical severity.

The four most common *AGXT* mutations in PH1 in European and North American patients are p.G170R, p.F152I, p.I244T and c.33_-_34insC [19,20]. The p.S205P mutation is a PH1-specific mutation in Japanese patients [21]. In the present study, mutational analysis of the *AGXT* gene in two Chinese families with PH1 revealed two patients who had compound heterozygous mutations. One patient had p.S81X and p.S275delinsRAfs mutations, and the other had p.M1T and p.I202N mutations. These four mutations are different from the four most common *AGXT* mutations in European and North American patients, which are also different from the PH1-specific mutation (p.S205P) in Japanese patients. The p.S275delinsRAfs and p.M1T mutations have been previously reported [22,23]. The p.M1T mutation was first reported in another Chinese study from Hong Kong in 2004 [23]. This mutation has not been reported in other populations to date. The other two mutations, p.S81X and p.I202N, are novel. The p.S81X mutation is a nonsense mutation that leads to a truncated AGT, and this mutation was speculated to be a pathogenic mutation. p.I202N is another missense mutation and is located on exon 6. Exon 6 spans the PLP co-factor binding site consensus sequence (amino acids 201–221) common to aminotransferases and is critical to the catalytic site [24]. Crystallisation studies confirmed that the lysine at codon 209 in exon 6 is the actual site of the Schiff base with PLP [25]. The alignment of AGT proteins from several species revealed that the isoleucine at codon 202 is 100% conserved across all analysed species (Figure 2d) and constitute one of the amino acids in the sequence of a highly conserved region of AGT protein. Several clinical reports identified nine mutations also located in this highly conserved sequence motif [6,21,26-29]. Given the predictions of the *in silico* PolyPhen-2 and SIFT analyses and the fact that the mutation was not identified in 100 healthy controls, the missense p.I202N mutation identified in patient 1 in this highly conserved sequence motif is hypothesised to be a damaging mutation. Therefore, the two novel mutations, p.S81X and p.I202N, were not identified in 100 healthy controls and are considered to be disease-causing mutations.

*AGXT* gene mutations result in deficiency and/or mistargeting of hepatic AGT, which leads to metabolic overproduction of oxalate and glycolate. The excess oxalate is excreted in the urine but is of low solubility and precipitates as a calcium salt, resulting in urolithiasis, nephrocalcinosis, and progressive renal insufficiency [11,30]. Both patients in this study presented with haematuria, back pain, and recurrent urolithiasis. Radiographic screening revealed multiple urolithiasis in both patients. Stone analysis revealed the main component of the stones to be calcium oxalate monohydrate (>95%). The patient with p.S81X and p.S275delinsRAfs mutations, presenting at the age of 3, did not respond to treatment with pyridoxine and progressed rapidly to end-stage renal disease (ESRD) at the age of 10.6. This patient displayed serious clinical features given that she had compound truncation mutations, which led to hepatic AGT deficiency. The patient with the p.M1T and p.I202N missense mutations presented at the age of 5 and received conservative treatment, including a high fluid intake, oral potassium citrate, and oral pyridoxine. Currently, her renal function is normal. PH1 may present at any age [4]. The presentation varies from infantile nephrocalcinosis and failure to thrive as a result of renal impairment to recurrent or occasional stone formation [30]. Patients should undergo metabolic screening for PH1 at the presentation of a first kidney stone (in a child), in recurrent or familial stone disease (at any age), or if nephrocalcinosis is detected [10,31]. Stone analysis may reveal characteristic morphology and whether the stone contains > 95% calcium oxalate monohydrate, which often presents with a particular morphology [32]. PH1 should be considered in any patient with renal failure of unknown cause, particularly in the presence of nephrocalcinosis or severe stone burden, because PH1 accounts for 1 to 2% of cases of paediatric ESRD according to registries from Europe, the United States, and Japan [33]. Radiographic screening of the kidneys may elucidate stones and/or medullary or diffuse nephrocalcinosis [30]. Although hepatic biopsy is the gold standard for PH1 diagnosis, it is invasive, and facilities for the estimation of enzyme activity are not available in many countries, especially developing countries. Hence, molecular diagnosis using direct sequencing of the whole *AGXT* gene is recommended. Therefore, the gold standard method of diagnosis—liver-biopsy-proven enzyme deficiency or enzyme mislocalisation—is now exclusively performed in patients for whom clinical suspicion is still evident but mutation analysis did not result in a precise diagnosis [2].

This is the first mutational analysis of PH1 performed in mainland China. Because facilities for enzyme estimation of liver biopsies are not available in most centres, there is a need to define clinical features and family histories of consanguinity. We believe that the findings of nephrocalcinosis, recurrent urolithiasis, and progressive renal failure in patients with severe stone burden are sufficient to clinically diagnose PH1. Detection of mutations in the *AGXT* gene may confirm the diagnosis of PH1.

## Conclusions

These are the first cases of primary hyperoxaluria type 1 to be diagnosed by clinical manifestations and *AGXT* gene mutations in mainland China. The novel p.S81X and p.I202N mutations detected in our study extend the spectrum of known *AGXT* gene mutations. The p.M1T mutation may be specific to Chinese patients.

## Competing interests

The authors declare that they have no competing interests.

## Authors’ contributions

HX and YA conceptualised the study. G-mL performed the experiment. QS and X-yF obtained funding. HX, G-mL, Y-nG, QS, X-yF, LS, and H-mL acquired the data. G-mL analysed the data. HX, YA, G-mL, and Y-nG contributed to data interpretation and manuscript preparation. All authors read and approved the final manuscript.

## Pre-publication history

The pre-publication history for this paper can be accessed here:

http://www.biomedcentral.com/1471-2369/15/92/prepub
